# General practitioners’ deprescribing decisions in older adults with polypharmacy: a case vignette study in 31 countries

**DOI:** 10.1186/s12877-020-01953-6

**Published:** 2021-01-07

**Authors:** Katharina Tabea Jungo, Sophie Mantelli, Zsofia Rozsnyai, Aristea Missiou, Biljana Gerasimovska Kitanovska, Birgitta Weltermann, Christian Mallen, Claire Collins, Daiana Bonfim, Donata Kurpas, Ferdinando Petrazzuoli, Gindrovel Dumitra, Hans Thulesius, Heidrun Lingner, Kasper Lorenz Johansen, Katharine Wallis, Kathryn Hoffmann, Lieve Peremans, Liina Pilv, Marija Petek Šter, Markus Bleckwenn, Martin Sattler, Milly van der Ploeg, Péter Torzsa, Petra Bomberová Kánská, Shlomo Vinker, Radost Assenova, Raquel Gomez Bravo, Rita P. A. Viegas, Rosy Tsopra, Sanda Kreitmayer Pestic, Sandra Gintere, Tuomas H. Koskela, Vanja Lazic, Victoria Tkachenko, Emily Reeve, Clare Luymes, Rosalinde K. E. Poortvliet, Nicolas Rodondi, Jacobijn Gussekloo, Sven Streit

**Affiliations:** 1 Institute of Primary Health Care (BIHAM), University of Bern, Bern, Switzerland; 2 Research Unit for General Medicine and Primary Health Care, Faculty of Medicine, School of Health Sciences, University of Ioannina, Ioannina, Greece; 3 Department of Nephrology and Department of Family Medicine, University Clinical Centre, University St. Cyril and Metodius, Skopje, Macedonia; 4 Institute for General Practice, University of Duisburg-Essen, University Hospital Essen, Essen, Germany; 5 Institute of General Practice and Family Medicine, University of Bonn, Bonn, Germany; 6 Primary, Community and Social Care, Keele University, Keele, Staffordshire ST5 5BG, United Kingdom; 7 Irish College of General Practitioners, Dublin, Ireland; 8 Hospital Israelita Albert Einstein, São Paulo, Brazil; 9 Family Medicine Department, Wroclaw Medical University, Wrocław, Poland; 10 Department of Clinical Sciences, Centre for Primary Health Care Research, Lund University, Malmö, Sweden; 11 Romanian Society of Family Medicine, Bucharest, Romania; 12 Department of Medicine and Optometry, Linnaeus University, Kalmar, Sweden; 13 Hannover Medical School, Center for Public Health and Healthcare, Hannover, Germany; 14 Danish College of General Practitioners, Copenhagen, Denmark; 15 Primary Care Clinical Unit, the University of Queensland, Brisbane, Australia; 16 Department of General Practice and Family Medicine, Center for Public Health, Medical University of Vienna, Vienna, Austria; 17 Department of Primary and Interdisciplinary Care, University Antwerp, Antwerp, Belgium; 18 Department of Nursing and Midwifery, University Antwerp, Antwerp, Belgium; 19 Department of Family Medicine, University of Tartu, Tartu, Estonia; 20 Department of Family Medicine, Medical Faculty, University of Ljubljana, Ljubljana, Slovenia; 21 Department of General Practice, Faculty of Medicine, University of Leipzig, Leipzig, Germany; 22 SSLMG, Societé Scientifique Luxembourgois en Medicine generale, Luxembourg City, Luxembourg; 23 Department of Public Health and Primary Care, Leiden University Medical Center, Leiden, the Netherlands; 24 Department of Family Medicine, Semmelweis University, Budapest, Hungary; 25 Department of Social Medicine, Charles University, Faculty of Medicine in Hradec Kralove, Hradec Kralove, Czech Republic; 26 Department of Family Medicine, Sackler Faculty of Medicine, Tel Aviv University, Tel Aviv, Israel; 27 Department of Urology and General Medicine, Faculty of Medicine, Medical University of Plovdiv, Plovdiv, Bulgaria; 28 Institute for Health and Behaviour, Research Unit INSIDE, University of Luxembourg, Luxembourg, Luxembourg; 29 Family Doctor, Invited Assistant of the Department of Family Medicine, NOVA Medical School, Lisbon, Portugal; 30 INSERM, Université de Paris, Sorbonne Université, Centre de Recherche des Cordeliers, Information Sciences to support Personalized Medicine, F-75006 Paris, France; 31 Department of Medical Informatics, Hôpital Européen Georges-Pompidou, AP-HP, Paris, France; 32 Family Medicine Department, Medical School, University of Tuzla, Tuzla, Bosnia and Herzegovina; 33 Faculty of Medicine, Department of Family Medicine, Riga Stradiņs University, Riga, Latvia; 34 Clinical Medicine, Faculty of Medicine and Health Technology, Tampere University, Tampere, Finland; 35 Dom zdravlja Zagreb - Centar, Zagreb, Croatia; 36 Department of Family Medicine, Institute of Family Medicine at Shupyk National Medical Academy of Postgraduate Education, Kyiv, Ukraine; 37 Quality Use of Medicines and Pharmacy Research Centre, UniSA: Clinical and Health Sciences, University of South Australia, Adelaide, South Australia Australia; 38 Geriatric Medicine Research, Faculty of Medicine and College of Pharmacy, Dalhousie University and Nova Scotia Health Authority, Halifax, NS Canada; 39 UWV (Employee Insurance Agency), Leiden, the Netherlands; 40 Department of General Internal Medicine, Inselspital, Bern University Hospital, University of Bern, Bern, Switzerland; 41 Department of Internal Medicine, Section Gerontology and Geriatrics, Leiden University Medical Center, Leiden, the Netherlands

**Keywords:** Deprescribing, Polypharmacy, Multimorbidity, Primary health care, Old age

## Abstract

**Background:**

General practitioners (GPs) should regularly review patients’ medications and, if necessary, deprescribe, as inappropriate polypharmacy may harm patients’ health. However, deprescribing can be challenging for physicians. This study investigates GPs’ deprescribing decisions in 31 countries.

**Methods:**

In this case vignette study, GPs were invited to participate in an online survey containing three clinical cases of oldest-old multimorbid patients with potentially inappropriate polypharmacy. Patients differed in terms of dependency in activities of daily living (ADL) and were presented with and without history of cardiovascular disease (CVD). For each case, we asked GPs if they would deprescribe in their usual practice. We calculated proportions of GPs who reported they would deprescribe and performed a multilevel logistic regression to examine the association between history of CVD and level of dependency on GPs’ deprescribing decisions.

**Results:**

Of 3,175 invited GPs, 54% responded (*N* = 1,706). The mean age was 50 years and 60% of respondents were female. Despite differences across GP characteristics, such as age (with older GPs being more likely to take deprescribing decisions), and across countries, overall more than 80% of GPs reported they would deprescribe the dosage of at least one medication in oldest-old patients (> 80 years) with polypharmacy irrespective of history of CVD. The odds of deprescribing was higher in patients with a higher level of dependency in ADL (OR =1.5, 95%CI 1.25 to 1.80) and absence of CVD (OR =3.04, 95%CI 2.58 to 3.57).

**Interpretation:**

The majority of GPs in this study were willing to deprescribe one or more medications in oldest-old multimorbid patients with polypharmacy. Willingness was higher in patients with increased dependency in ADL and lower in patients with CVD.

**Supplementary Information:**

The online version contains supplementary material available at 10.1186/s12877-020-01953-6.

## Background

Polypharmacy, commonly defined as the concurrent use of 5 or more medications, is a growing concern in a context of common overtreatment. More than 40% of older adults aged 65 years and over and an even higher percentage of older nursing home residents have polypharmacy [1, 2]. Polypharmacy can be problematic as it is associated with a higher risk of being prescribed potentially inappropriate medications (PIMs) [3]. One third of adults aged 65 years and over are taking at least one PIM [4]. Polypharmacy and PIMs are linked to an increased risk of adverse drug events [5, 6], drug-drug and drug-disease interactions [7, 8], functional decline [9–11], decline in cognitive function [10, 12], increased risk for falls [13, 14], and increase in direct medical healthcare costs [15].

Older multimorbid adults with cardiovascular diseases (CVD) have been shown to be disproportionately affected by medication-related issues [16]. Due to these potential negative consequences optimizing polypharmacy in older adults including those with CVD is highly relevant.

With increasing age the main treatment goals often shift from the prevention of mortality and morbidity to the maintaining of functional independence and quality of life, especially in less robust older adults with limited levels of independence [17]. In addition, the benefit-risk profile of older dependent and less robust adults is altered as they are at greater risk of medication induced harm and may not have sufficient remaining life span to benefit from preventive medications [18, 19]. Therefore, older adults with limited functional independence might particularly benefit from medication optimization through deprescribing. However, little is currently known about general practitioners’ (GPs) attitudes towards deprescribing in patients with and without history of cardiovascular disease or in those with limited functional independence.

In recent years, deprescribing has become a popular “new word to guide medication review” [20]. It is commonly defined as ‘the process of withdrawal or [reduction] of an inappropriate medication, supervised by a healthcare professional with the goal of managing polypharmacy and improving outcomes’ [21]. Deprescribing has several benefits, such as achieving better health outcomes through resolving adverse drug reactions, better medication adherence, and direct medical healthcare costs reductions [22]. However, deprescribing may also have negative consequences, such as withdrawal reactions and the worsening or return of medical conditions. These potential harms can be minimized with appropriate planning, monitoring, and re-initiation of medications if needed [22]. As evidenced by the high prevalence of inappropriate medication use in older adults, deprescribing is not routinely conducted in practice. Despite its potential benefits, deprescribing is difficult to implement [23]. In practice, both physicians and patients report barriers to deprescribing, such as uncertainty on how to deprescribe due to a lack of evidence-based guidelines. Patients have reported believing that their medications are still necessary or beneficial [24–27]. An understanding of GPs’ deprescribing decisions and the potential barriers they face is needed to inform GP education and develop interventions to optimise appropriate medication use in older adults.

In a case vignette study with 157 GPs in Switzerland, we found a high rate of hypothetical deprescribing of certain medications, which was influenced by patients' history of CVD [28]. However, we were not able to establish the generalisability of these results and the influence of other patient characteristics on GPs deprescribing decisions. Therefore, the aim of this study was to examine deprescribing decisions of GPs in oldest-old patients (80 years and over) with polypharmacy across different countries and to examine whether increasing levels of dependency in activities of daily living (ADL) and history of CVD influenced these decisions.

## Methods

### Setting and study design

**Fig. 1:**
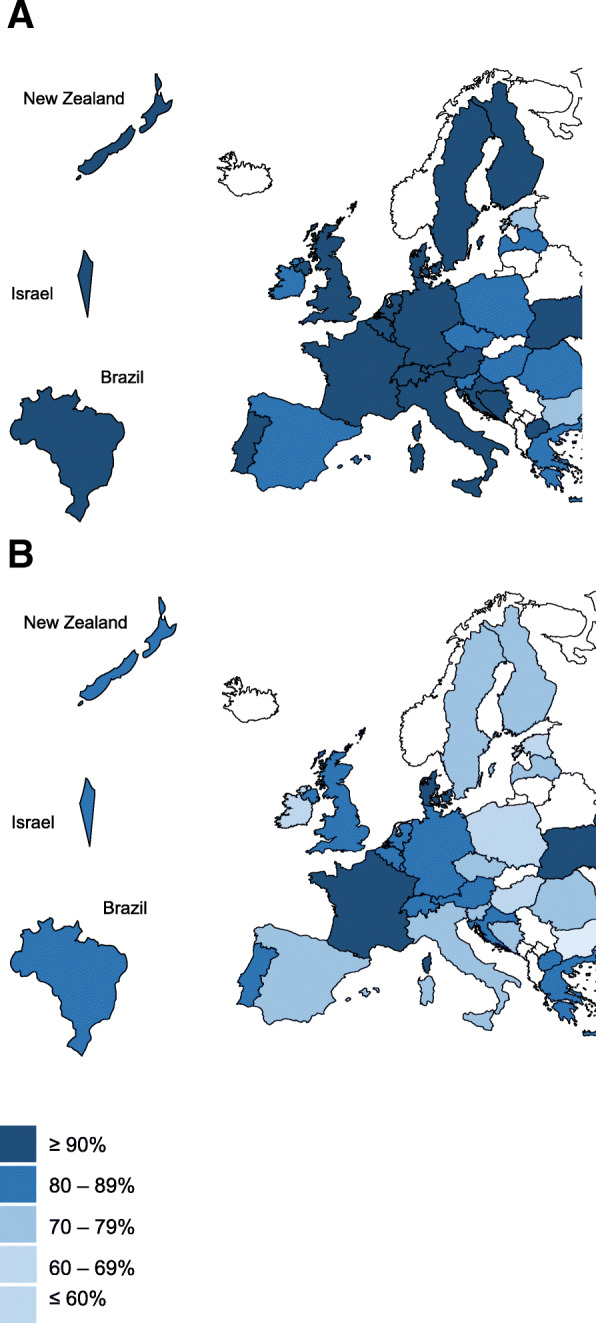
Per country average of the percentage of case vignettes in which GPs (*N* = 1,706) reported they would deprescribe at least one (map A) vs. at least two (map B) medications. List of participating countries (alphabetical order): Austria, Belgium, Bosnia and Herzegovina, Brazil, Bulgaria, Croatia, Czech Republic, Denmark, United Kingdom, Estonia, Finland, France, Germany, Greece, Hungary, Ireland, Israel, Italy, Latvia, Luxembourg, Macedonia, the Netherlands, New Zealand, Poland, Portugal, Romania, Slovenia, Spain, Sweden, Switzerland, Ukraine. Maps designed by and adapted from PresentationGO.com /* © Copyright* PresentationGO.com

This is a cross-sectional case vignette study conducted with GPs from 31 countries (see Fig. 1). It is part of the LESS (barriers and enabLers to willingnESs to depreScribing in older patients with multimorbidity and polypharmacy and their General Practitioners) study.

### Participants

Our total sample consisted of 3,175 GPs from 31 countries who were invited to participate by email through national coordinators. Participants had previously provided consent to be contacted with opportunities to participate in future research [29, 30]. Participants were eligible for inclusion if they were practicing GPs.

### Questionnaire

We used the same questionnaire as described in Mantelli et al. (2018), but we included additional case vignettes [28]. We used the *Checklist for Reporting Results of Internet E-Surveys* (CHERRIES) guidelines for reporting results of internet e-surveys [31, 32]. The questionnaire had 3 parts: 1) GP characteristics, 2) 3 case vignettes of oldest-old patients with higher/heightened dependency in activities of daily living (ADL) including increasing cognitive impairment, each presented with and without history of CVD, and 3) Likert-scale questions concerning factors influencing GPs’ deprescribing decisions. For the complete questionnaire, refer to Additional file 1: Appendix 1. Where necessary, national coordinators translated and back-translated the survey from English into 22 languages. In Finland and Israel, the survey was distributed in English. In all other countries the survey was distributed in one or several national languages (see Additional file 1: Appendix 2 for more information on survey languages). The online survey was distributed and administered with SurveyMonkey (Palo Alto, CA, USA).

To sample the participating GPs, first, we engaged with national coordinators through the European General Practice Research Network (EGPRN). Second, national coordinators identified relevant networks through which the survey could be distributed. Available networks varied depending on the country. Most national coordinators did a convenience sampling in which they distributed the survey by email to GPs in their personal networks, who had previously consented to be invited to participate in research. Participation was voluntary. In some countries, the survey was sent to lists of GPs available at primary care research institutes or professional societies, which explains the bigger sample size in these countries. Reminders were used when necessary (maximum two reminders where sent). The response rate for each country can be found in Additional file 1: Appendix 2. In Ukraine the survey was administered on paper during a national GP conference due to infrastructure-related reasons. We collected responses from February to December 2018.

Our research team, largely composed of GPs, designed the case vignettes with the aim of creating hypothetical patients aged ≥80 years representing patients typically seen in primary care. Repeated meetings to discuss the case vignettes were held. Collaborators in other countries were consulted by email, with changes made as necessary. Before starting the data collection, the online questionnaire was piloted among five Swiss GPs to test its content validity. Before starting the data collection in each participating country, each national coordinator checked and, if applicable, adapted the layout of the survey based on the local context.

The case vignettes were identical except for CVD status and levels of dependency in ADL. We provided descriptions of dependency related to low, medium and high impairment of ADL and cognitive function. All hypothetical patients were prescribed the same medications. For every case vignette, we asked GPs whether they would stop/reduce the dosage of at least one medication (i.e. deprescribe), and if so which one(s). GPs were instructed to respond as to how they would act in their usual practice.

In part 3 of the questionnaire, GPs were asked to rate the importance of sixteen factors that potentially influenced their deprescribing behaviour using 5-point Likert-scales ranging from “not important” to “very important”. The selection of these factors was based on work done by Luymes et al. [33] and Anderson et al. [34] and was completed with factors based on our team’s experience.

Completion of the survey took 10–15 minutes. The different parts of the questionnaire were presented on different pages and where necessary the content of one part was distributed over different pages to keep the number of items per page small. Respondents were able to navigate back and forth through the survey. The national coordinators sent a web link to GPs, which was required to access the survey. The selection of one response option was enforced. We did not use cookies nor did we collect IP addresses. We did not perform a timestamp analysis.

### Statistical analyses

We described GP characteristics by calculating proportions, means, and confidence intervals (CI). We calculated crude odds ratios (OR) from univariate logistic regressions to determine if GP characteristics were associated with decisions to deprescribe. For each case vignette we described the proportions of GPs who would deprescribe. As a sensitivity analysis, we also performed this analysis in countries with a > 60% response rate. We calculated the average number of medications deprescribed per case vignette. We performed a multilevel logistic regression to examine the association between both history of CVD and level of dependency in ADL and GPs’ decisions to deprescribe at least one medication in any of the case vignettes by accounting for the clustering of GPs at country level. We adjusted the model for the following GP characteristics: age, sex, average number of consultations per day, frequency of seeing patients with polypharmacy. Subsequently, we performed a comparison of proportions to determine whether GPs’ deprescribing decisions concerning specific medications changed with increased patient dependency. Lastly, for the factors included in the Likert-scales we calculated the percentage of GPs who rated these factors as (very) important. We defined a two-sided *p*-value of < 0.05 as significant. All analyses were performed with STATA 15.1 (StataCorp, College Station, TX, USA).

## Results

### GP characteristics

In the participating countries, the median response rate was 50% (range: 11–95%). Of the total of 3,175 invited GPs across countries, 1,706 responded (54%), and 1415 GPs completed the whole questionnaire. The number of participants differed by country (range: 20 in Czech Republic and Ireland; 247 in Hungary).

Table 1 presents characteristics of the participating GPs. 60% were female, mean age was 50 years, and the mean clinical experience as GP was 18 years. As shown in this table, being female reduced the odds of deprescribing in all case vignettes (compared to not deprescribing in one or more case vignettes), whereas the odds of deprescribing increased with increasing age of GPs, with GPs regularly treating patients aged 70 years or more with polypharmacy and with GPs regularly dealing with the topic of deprescribing.

**Table 1:** Baseline characteristics of general practitioners (GPs) from all participating countries (N countries = 31, N GPs = 1,706)

		GPs’ deprescribing decisions^a^ (*N* = 1,428, only complete records)			
GP characteristics	Overall	Deprescribing in < 6 case vignettes (*n* = 370)	Deprescribing in all 6 case vignettes^b^ (*n* = 1,058)	Crude odds ratio of deprescribing in all 6 case vignettes^c^ (95% CI)	*P*-value^d^
*Sex*					
female, n (%)	1,021 (60)	240 (65)	593 (56)	0.74 (0.57 to 0.96)	0.024
male, n (%)	685 (40)	130 (35)	465 (44)	ref.	
*Age, in years*					
*mean (standard deviation)*	50 (12)	49 (12)	50 (12)	*per 10 years*: 1.14 (1.02 to 1.28)	0.020
*Clinical experience as GP, in years*					
*mean (standard deviation)*	18 (11.4)	17 (11)	18 (11)	*per 10 years:* 1.12 (1.00 to 1.25)	0.055
*Average number of consultations per working day, n (%)*					
< 15	197 (12)	31 (8)	121 (11)	ref.	–
15–25	567 (33)	123 (33)	356 (34)	0.78 (0.48 to 1.25)	0.30
26–35	468 (27)	93 (25)	300 (28)	0.91 (0.56 to 1.50)	0.72
> 35	474 (28)	123 (33)	281 (27)	0.71 (0.43 to 1.20)	0.21
*Frequency of seeing/treating patients aged ≥ 70 years with polypharmacy, n (%)*					
frequently / very frequently	1,469 (87)	310 (84)	942 (89)	1.63 (1.15 to 2.32)	0.006
very rarely / rarely / occasionally	218 (13)	60 (16)	116 (11)	ref.	–
*Frequency of dealing with the topic of deprescribing medications in daily practice, n (%)*					
frequently / very frequently	935 (56)	176 (48)	638 (60)	1.53 (1.18 to 1.97)	0.001
very rarely / rarely / occasionally	729 (44)	194 (52)	420 (40)	ref.	–
*Frequency of deprescribing medications during consultations in daily practice, n (%)*					
frequently / very frequently	438 (26)	76 (21)	305 (29)	1.46 (1.09 to 1.97)	0.012
very rarely / rarely / occasionally	1,226 (74)	294 (79)	753 (71)	ref.	-

^a^deprescribing defined as stopping or reducing the dosage of at least one medication; ^b^median deprescribing behaviour corresponds to deprescribing or reducing the dosage of at least one medication in all of the 6 hypothetical patients; ^c^crude odds ratios from multilevel univariate logistic regression; ^d^*P*-values from univariate logistic regression

### Deprescribing decisions

Table 2 shows the percentage of GPs reporting stopping at least one, two or three medications per case vignette. More than 90% (range: 94–95%) of GPs reported that they would deprescribe at least one medication in all the case vignettes without history of CVD whereas the proportion was slightly lower (range: 82–90%) in the case vignettes with history of CVD. Around 70% of GPs (range: 68–78%) opted for deprescribing at least 3 medications in the case vignettes without CVD history while the percentage again was lower (range: 27–59%) in the case vignettes with CVD history. In CVD cases, the proportion of GPs who reported deprescribing medications increased with increasing dependency levels. The sensitivity analysis performed in countries with a response rate > 60% showed the same trends (Additional file 1: Appendix 3).

**Table 2:** Percentage of general practitioners (GPs) deprescribing in case vignettes, sorted by GPs’ decisions to deprescribe at least one, two or three medications in the respective case vignette, patients’ level of dependency in activities of daily living, and patients’ history of cardiovascular disease (CVD) (*N* = 1,706)

Case vignette	Patients’ dependency level	Deprescribing decision	Without history of CVD (95% CI)	With history of CVD (95% CI)	Difference (95% CI)^a^
1	low				
	(living in own house, no help needed for activities of daily living)	min. 1 medication	95.1% (94.0 to 96.1)	81.6% (79.6 to 83.5)	13.5% (11.3 to 15.7)
		min. 2 medications	88.2% (86.6 to 89.8)	60.1% (57.7 to 62.5)	28.1% (25.2 to 31.0)
		min. 3 medications	69.2% (66.9 to 71.5)	26.5% (24.3 to 28.7)	42.7% (39.6 to 45.9)
2	medium				
	(living in own house, some help needed for activities of daily living)	min. 1 medication	94.3% (93.1 to 95.5)	87.4% (85.7 to 89.1)	6.8% (4.8 to 8.9)
		min. 2 medications	85.8% (84.0 to 87.5)	68.5% (66.1 to 70.9)	17.3% (14.3 to 20.3)
		min. 3 medications	67.6% (65.3 to 70.0)	36.6% (34.1 to 39.1)	31.0% (27.6 to 34.5)
3	high				
	(living in nursing home, help needed for nearly all activities of daily living)	min. 1 medication	94.1% (92.8 to 95.3)	90.4% (88.8 to 91.9)	3.7% (1.7 to 5.7)
		min. 2 medications	88.5% (86.8 to 90.1)	79.2% (77.1 to 81.3)	9.3% (6.6 to 12.0)
		min. 3 medications	78.4% (76.2 to 80.5)	58.6% (56.0 to 61.1)	19.8% (16.5 to 23.1)

^a^Two-sample test of proportions

The multilevel logistic regression model of GPs’ decisions to deprescribe at least one medication in any case vignette, adjusted for GP characteristics, showed that the odds of GPs reporting deprescribing in patients without CVD history were 3 times higher than the odds of GPs reporting to deprescribe in patients with history of CVD (Table 3). The odds of GPs reporting deprescribing in the scenarios with an increased level of dependency were 1.29 to 1.50 times higher than the odds of GPs reporting deprescribing in the scenarios in which patients had lower dependency levels. While GPs’ age was associated with taking deprescribing decisions (OR: 1.14 for 10-year increase, 95% CI: 1.06–1.23), female sex was not (OR: 0.89, 95% CI: 0.75–1.05) nor were the average number of consultations per day or the frequency of seeing patients with polypharmacy (Table 3).

**Table 3:** Multilevel logistic regression model: adjusted effect of patient and general practitioners’ (GPs) characteristics on general practitioners’ decisions to deprescribe at least one medication in any of the case vignettes (N = 1,706)

	Overall		
	*Odds ratio*	*95% confidence interval*	*P-value*
*Patient’s history of cardiovascular disease (CVD)*			
History of CVD	ref.	–	–
No history of CVD	3.04	2.58 to 3.57	< 0.001
*Patient’s level of dependency in activities of daily living*			
Low	ref.	-	-
Medium	1.29	1.09 to 1.55	0.004
High	1.50	1.25 to 1.80	< 0.001
*Age (GP), 10-year increase*	1.14	1.06 to 1.23	< 0.001
*Female sex (GP)*	0.89	0.75 to 1.05	0.167
*Number of consultations per day*			
< 15	ref.	–	–
15–25	1.04	0.77 to 1.40	0.79
26–35	1.2	0.88 to 1.65	0.25
> 35	0.94	0.68 to 1.30	0.698
*Frequency of seeing patients with polypharmacy*			
Never	ref.	–	–
Rarely	0.64	0.18 to 2.28	0.497
Occasionally	0.80	0.25 to 2.53	0.699
Frequently	1.27	0.39 to 3.87	0.728
Very frequently	1.42	0.45 to 4.49	0.554

The multilevel model accounts for clustering of the GPs at country level

### Geographical variation

Figure 1 maps the differences in the per country averages of case vignettes in which GPs from our convenience sample opted for deprescribing in at least one versus at least two medications. The percentages of deprescribing a minimum of one medication ranged from 77% in Bulgaria to 100% in Ukraine, whereas the percentages of deprescribing a minimum of two medications ranged from 58% in Bulgaria to 92% in Denmark. Both maps show variation across countries.

### Deprescribing decisions by medication type

Table 4 shows the proportion of GPs who would deprescribe sorted by medication type, CVD history, and level of dependency in ADL. There was little variation in reported deprescribing for pantoprazole, tramadol, and paracetamol among the different levels of dependency and CVD history. For atorvastatin, aspirin, amlodipine, and enalapril the percentages of GPs reporting to deprescribe generally increased with increasing levels of dependency and was lower when there was a history of CVD. Overall, GPs were most likely to deprescribe proton-pump inhibitors and pain medication.

**Table 4:** Comparison of crude percentages of general practitioners (GPs) reporting to deprescribe the medications in the case vignettes, sorted by medication type, history of cardiovascular disease (CVD), and dependency level (*N* = 1,706)

Medication	Level of dependency in activities of daily living		
	***Low*** ***(case vignette 1)***	***Medium*** ***(case vignette 2)***	***High*** ***(case vignette 3)***
	Percentage of GPs (95% CI)	Percentage of GPs (95% CI)	Percentage of GPs (95% CI)
*Pain medications*			
**Tramadol 50 mg, twice daily**			
Without history of CVD	63.5% (61.1 to 65.9)	69.4% (67.0 to 71.7)	68.5% (66.0 to 70.9)
With history of CVD	57.3% (55.2 to 60.2)	67.0% (64.5 to 69.4)	67.6% (65.2 to 70.1)
**Paracetamol 1 g, three times daily**			
Without history of CVD	47.5% (45.0 to 50.0)	41.9% (39.4 to 44.5)	44.9% (42.3 to 47.5)
With history of CVD	43.8% (41.3 to 46.3)	40.8% (38.3 to 43.4)	43.6% (41.0 to 46.2)
*Proton-pump inhibitor*			
**Pantoprazole 20 mg, once daily**			
Without history of CVD	64.5% (63.0 to 67.8)	64.4% (61.9 to 66.8)	67.8% (65.3 to 70.2)
With history of CVD	47.1% (44.6 to 49.6)	49.0% (46.4 to 51.6)	55.6% (53.0 to 58.2)
*Antihypertensive medications*			
**Amlodipine 5 mg, once daily**			
Without history of CVD	15.2% (13.4 to 17.0)	18.9% (17.0 to 21.0)	33.9% (31.4 to 36.4)
With history of CVD	8.7% (7.3 to 10.2)	15.1% (13.3 to 17.1)	30.3% (27.9 to 32.8)
**Enalapril 10 mg, once daily**			
Without history of CVD	7.7% (6.4 to 9.1)	9.8% (8.3 to 11.4)	19.4% (17.4 to 21.5)
With history of CVD	2.5% (1.7 to 3.4)	4.6% (3.6 to 5.8)	15.5% (13.6 to 17.5)
*Cholesterol-lowering medication*			
**Atorvastatin 40 mg, once daily**			
Without history of CVD	59.1% (56.6 to 61.5)	62.7% (60.2 to 65.2)	76.8% (74.5 to 78.9)
With history of CVD	13.7% (12.0 to 15.5)	26.5% (24.3 to 28.9)	52.5% (49.9 to 55.1)
*Antiplatelet medication*			
**Aspirin 100 mg, once daily**			
Without history of CVD	52.1% (49.6 to 54.5)	49.1% (46.5 to 51.7)	60.3% (57.7 to 62.8)
With history of CVD	4.3% (3.4 to 5.5)	7.2% (5.9 to 8.6)	23.9% (21.7 to 26.2)

Acronyms: *CI* Confidence interval; *CVD* Cardiovascular disease; *GP* General practitioner

### Factors important for deprescribing decisions

**Fig. 2:**
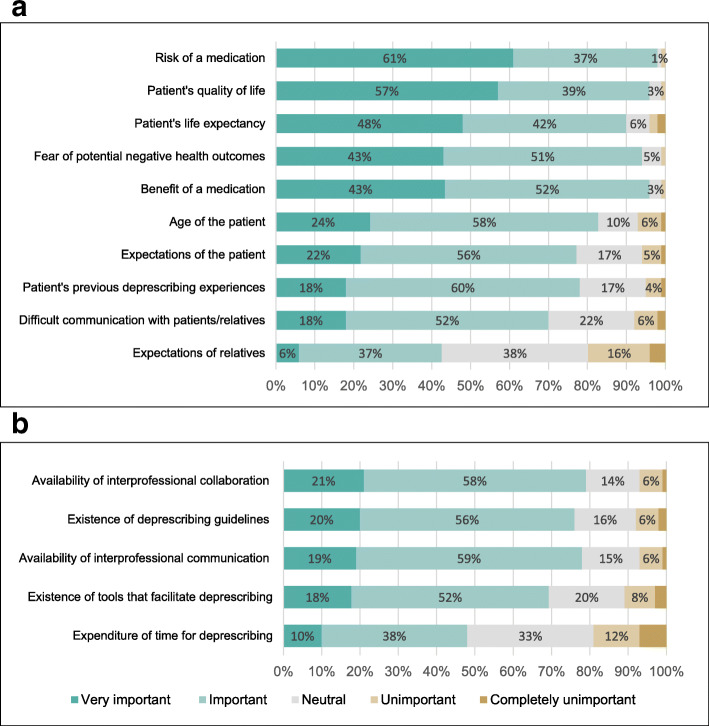
Factors important to general practitioners (GPs) when making deprescribing decisions^1^, ordered by importance (N = 1,706). a) factors related to the patient, and b) factors related to the GP. ^1^each GP was asked to rate the importance of each factor

Figure 2 shows the importance given to different factors reported to impact GPs’ deprescribing decisions. Risks and benefits of medications, patients’ quality of life, patients’ life expectancy and patients’ fear of potential negative health outcomes were important or very important to more than 90% of GPs. Less than half of GPs rated the time needed for deprescribing as important or very important for making deprescribing decisions.

## Discussion

In this study of over 1,700 GPs from 31 countries, we investigated GPs’ deprescribing decisions in oldest-old patients with polypharmacy. Despite differences across GP characteristics and across countries, a large proportion of GPs reported that they would deprescribe at least one medication in all scenarios. The odds of GPs reporting decisions to deprescribe was higher in patients with a higher dependency level (OR =1.5, 95%CI, 1.25 to 1.80) and in absence of CVD history (OR =3.04, 95%CI 2.58 to 3.57). The medications GPs were most willing to deprescribe in case vignettes with and without history of CVD were pain medications and proton-pump inhibitors. However, history of CVD appeared to affect deprescribing decisions of certain medications. While GPs were likely to deprescribe cholesterol medication used for primary prevention (no history of CVD), GPs were less likely to deprescribe those medications when used for secondary prevention. Factors GPs rated as important or very important for deprescribing decisions were patients’ quality of life, life expectancy, fear of potential negative health outcomes resulting from deprescribing, and the risks and benefits of medications.

This is the first study to examine deprescribing decisions of GPs across a large number of countries. We found variation in deprescribing decisions across countries and based on GP characteristics, such as age with older GPs being more likely to take deprescribing decisions. Bolmsjö et al. (2016) found that deprescribing behaviours were largely dependent on the structure of healthcare systems [35]. This might explain the differences we found between countries. Previous qualitative studies reported that GPs with greater clinical experience were more able to draw on their own clinical knowledge [36–39], which might explain why older and more experienced GPs in our sample were more likely to deprescribe. Further research is needed to explore the association between GP characteristics and deprescribing in more depth.

Our findings show that GPs were willing to deprescribe in patients with high dependency and increasing cognitive impairment. The results built on a first analysis with the Swiss data from the LESS study, in which we had only included the most dependent, least robust oldest-old adults (case vignette 3) and found that GPs reported to be influenced by the risk and benefit of medications, quality of life and life expectancy when taking deprescribing decisions [28]. Our findings are in line with previous research, which revealed cognitive impairment as an important factor for deprescribing [40]. This also aligns with the basic principles of appropriate medication use which contend that potential benefits of the medication should outweigh potential risks and align with the goals of care of the individual [19]. As mentioned before, the benefit-risk profile of dependent and less robust older adults is altered as they are at greater risk of medication induced harm and may not have sufficient remaining life span to benefit from preventive medications [18, 19]. That GPs seem more willing to deprescribe in older adults with increased dependency levels implies that we need better ways to identify such patients in primary care settings. The routine use of frailty screening tools in primary care is gaining interest. However, it remains unclear which tools are the most useful and feasible and how to best deliver care for those identified as frail and less robust [41, 42]. Furthermore, despite the fact that certain tools exist to conduct deprescribing in older adults with frailty or limited life expectancy, little is known about how such tools can be used in a way that reduces inappropriate medication use and improves clinical outcomes [43].

In line with a qualitative study by Luymes et al., we found that GPs were more likely to deprescribe in patients with a lower CVD risk [33]. A recent national cross-sectional survey of US geriatricians, general internists, and cardiologists found that > 90% of physicians in each specialty reported to deprescribe cardiovascular medications when patients experienced adverse drug reactions [44]. In addition, this study also pointed out potential barriers linked to the communication between physicians when making deprescribing decision. Our finding of the impact of CVD on deprescribing, however, is likely driven by the fact that four out of the seven medications in the case vignette are related to the cardiovascular system. Further research is warranted to find ways to overcome the barriers linked to inter-professional communication, as this is crucial for sustainable deprescribing.

The medications presented in our case vignettes are commonly used in older adults. However, some of them are considered potentially inappropriate to be used in older adults. For instance, according to the 2019 Beers criteria aspirin should not be used for primary prevention of cardiovascular disease, tramadol should be used with caution as it may cause or exacerbate the syndrome of inappropriate secretion of antidiuretic hormone, and the use of proton pump inhibitors for more than eight weeks should be avoided in non-high-risk patients [45]. In this study, GPs were most likely to opt for deprescribing proton pump inhibitors and pain medication in case vignettes with and without history of CVD while they were least likely to deprescribe antihypertensive medications. GPs were also likely to deprescribe aspirin and atorvastatin for primary prevention. This shows that GPs in our sample were likely to opt for deprescribing medications that are potentially inappropriate when used in older adults. This awareness needs to be built upon when shifting deprescribing from theory to practice. Generally reported deprescribing was high among the GPs when considering the medications as a whole. However, the results for aspirin show that there remain barriers to deprescribing even in hypothetical scenarios. In 2018 three large studies established that aspirin for primary prevention of CVD has a greater risk of harm and shows relatively modest benefits in relation to cardiovascular outcomes [46–48]. Therefore it would be interesting to see whether our study would yield different results (pertaining to aspirin) if it was repeated.

Further research is needed to create thorough guidance on how to deprescribe in older adults with potentially inappropriate polypharmacy, which includes studying the safety of deprescribing in this population group and to further investigate patient barriers to deprescribing [28]. Over 70% of GPs in our study perceive the existence of deprescribing guidelines and tools that facilitate deprescribing as important or very important. This underscores the need for creating such guidelines, not just on when to deprescribe but also how to deprescribe. It also points to a need to raise awareness of currently existing guidelines and potential benefits of translating guidelines to local languages. Currently, evidence-based deprescribing clinical practice guidelines exist for proton pump inhibitors, benzodiazepines and Z-drugs, antihyperglycemics, antipsychotics and cholinesterase inhibitors and memantine [49–53]. Furthermore, an in-depth exploration into the nuanced reasons why GPs do or do not deprescribe specific medications in specific situations and into how deprescribing could be sustainably implemented will be useful for improving deprescribing practices and guidelines.

Our study is strengthened by the inclusion of a large number of GPs from many different countries in Europe and beyond, some of which are rarely included in studies among GPs. Furthermore, the average response rate of 53% is higher than typical response rates of 30–40% in surveys among GPs [54]. The LESS study comes with several limitations. The first one is the hypothetical nature of our case vignettes, which were intended to establish and correspond to GPs’ routine clinical practice [28]. However, we were not able to capture the decision-making process, including barriers and facilitators of deprescribing, such as time limitations and patient preferences, values or goals of care, or capture the reasons why GPs selected to deprescribe or not. Therefore, the results of this study may not reflect the complex process of shared decision making. That said, the simple nature of the hypothetical case vignettes is also a strength, as it allowed gathering of a large number of responses from GPs in standardized cases. Second, we do not know how reported deprescribing decisions would transfer to other medications not included in the case vignettes. Third, we did not randomly sample the GPs in each country but performed a convenience sample based on the networks of our national coordinators, which comes with limited generalizability of our study results. Despite this, to maximise the number of countries involved in order to increase generalisability by reaching a larger number of GPs, we allowed for variations in the types of networks that national co-ordinators used to recruit participants. The variation in the types of networks used was also reflected in the large variation in response rates by country. In addition, GPs self-selecting to complete the survey were likely to be more interested in deprescribing, which may mean that our results could be biased towards overestimating deprescribing decisions. Fourth, we were limited to the self-reported data about GPs’ deprescribing decisions, which might have been affected by social desirability bias and the order in which case vignettes were presented. Fifth, we do not know to what extent the reported deprescribing decisions reflect or were influenced by national deprescribing guidelines or other deprescribing initiatives.

## Conclusions

Despite international variation, most GPs in our convenience sample reported they would deprescribe at least one medication in hypothetical oldest-old multimorbid patients with polypharmacy. Older GPs were more likely to take deprescribing decisions. GPs were more likely to deprescribe in patients with a higher dependency in activities of daily living and in the absence of a history of cardiovascular disease. Overall, medications most often chosen for deprescribing in the presented case vignettes were proton pump inhibitors and pain medications. Antiplatelet and cholesterol-lowering medication was frequently selected for deprescribing when used for primary prevention.

## Supplementary Information

**Additional file 1.** Appendix 1-3.

## Data Availability

The dataset used and analysed during the current study is available from the corresponding author on reasonable request.

## Abbreviations

ADL: Activities of daily living
CHERRIES: Checklist for Reporting Results of Internet E-Surveys
CI: Confidence interval
CVD: Cardiovascular disease
EGPRN: European General Practice Research Network
GPs: General practitioners
LESS: barriers and enabLers to willingnESs to depreScribing in older patients with multimorbidity and polypharmacy and their General Practitioners
OR: Odds ratio
PIM: Potentially inappropriate medication
