# Supplementary material for: General practitioners’ deprescribing decisions in older adults with polypharmacy: a case vignette study in 31 countries
Source: BMC Geriatr. 2021 Jan 7;21:19. doi: 10.1186/s12877-020-01953-6 (PMC7792080; doi:10.1186/s12877-020-01953-6)
Supplement: Supplementary file 1 — Additional file 1. Appendix 1-3. [file 12877_2020_1953_MOESM1_ESM.docx]

***General Practitioners’ Deprescribing Decisions in Older Adults with Polypharmacy: a Case Vignette Study in 31 Countries***

Jungo et al.

*Supplementary Material*

***Appendix 1.***

**GP Questionnaire used in the “barriers and enabLers to willingnESs to depreScribing in older patients with multimorbidity and polypharmacy and their General Practitioners” (LESS) study**

**A) GP background information**

1. Please indicate your age (in number of years).

2. Please indicate your sex. (male/female)

3. How many years have you been working as GP? (in number of years)

4. How many clinical consultations do you have on average per working day? (An average working day is a full day/2 sessions in the practice). (<15, 15-25, 26-35, >35)

5. How often do you see/treat patients who fulfil the following criteria:

- aged ≥70 years
- ≥3 chronic conditions
- ≥5 regular medications

(never, rarely, occasionally, frequently, very frequently)

Thinking of the patients who fulfil these three criteria. How would you answer the following questions?

- aged ≥70 years
- ≥3 chronic conditions
- ≥5 regular medications

6. How often do you deal with the topic of deprescribing medications in your daily practice with these patients? (never, rarely, occasionally, frequently, very frequently)

7. How often do you deprescribe medications during consultations with your patients in your daily practice in respect of these patients? (never, rarely, occasionally, frequently, very frequently)

**B) Case vignettes**

***Case vignette 1***

**Patient 1**, 82 years of age:

**Social history**: retired carpenter, *lives with his wife in a single-family home. Patient 1 prepares his medication independently, goes grocery shopping and does other work around the house and garden. The couple do not require any help from third parties.*

**General health**: *in a good physical and cognitive condition. MMSE 28/30.*

**Other diagnoses**: chronic back pain, hypertension, non-smoker, no past history of cardiovascular events, no family history of cardiovascular events

**Laboratory values**: dyslipidemia (3.8mmol/l), liver and kidney function are normal (taking into account the age of the patient), normal blood count. Last systolic blood pressure measurements ranged from 130 to 140mmHg.

**Daily medication intake:**

Aspirin 100 mg once daily

Atorvastatin 40 mg once daily

Enalapril 10 mg once daily

Amlodipine 5 mg once daily

Paracetamol 1 g three times a day

Tramadol 50 mg twice daily

Pantoprazole 20mg once daily

**In this case vignette, you consider the patient:**

- to have a good physical functioning and somatic condition

- to be totally independent

- to be cognitive not impaired

- to have a low risk of cardiovascular events

8. Would you deprescribe or decrease the dosage of one/several medication/s? (yes/no)

9. Which medication/s would you deprescribe or decrease?

- - Aspirin 100 mg once daily
  - Atorvastatin 40 mg once daily
  - Enalapril 10 mg once daily
  - Amlodipine 5 mg once daily
  - Paracetamol 1g three times a day
  - Tramadol 50 mg twice daily
  - Pantoprazole 20 mg once daily

10. Consider that Patient 1 now had a cardiovascular event in the past (e.g. myocardial infarction three years ago). Would you deprescribe or decrease the dosage of one/several medication/s? (yes/no)

11. Which medication/s would you deprescribe or decrease taking into account that Patient 1 has already had a cardiovascular event in the past (e.g. myocardial infarction three years ago)?

- - Aspirin 100 mg once daily
  - Atorvastatin 40 mg once daily
  - Enalapril 10 mg once daily
  - Amlodipine 5 mg twice daily
  - Paracetamol 1g three times a day
  - Tramadol 50 mg twice daily
  - Pantoprazole 20 mg once daily

***Case vignette 2***

**Patient 2**, 82 years of age:

**Social history:** retired carpenter, *lives with his wife who is in a good physical and cognitive state. Patient 2 is becoming more and more dependent; household tasks are done by his wife. Patient 2 needs help from third parties for personal hygiene, getting dressed/undressed and preparing medication.*

**General state**: walking pace significantly decreased over the past year, unsteady on his legs. Increasing forgetfulness and attention deficiency in the past couple of months. MMSE 22/30.

**Other diagnoses:** Chronic back pain, hypertension, non-smoker, no past history of cardiovascular events, no family history of cardiovascular events

**Laboratory values**: Dyslipidemia (LDL 3,8mmol/l), liver and kidney function are normal (taking into account the age of the patient), normal blood count. Last systolic blood pressure measurements ranged from 130 to 140mmHG.

**Daily medication intake:**

Aspirin 100 mg once daily

Atorvastatin 40 mg once daily

Enalapril 10 mg once daily

Amlodipine 5 mg once daily

Paracetamol 1 g three times a day

Tramadol 50 mg twice daily

Pantoprazole 20mg once daily

**In this case vignette, you consider the patient:**

- to have reduced physical functioning

- to be increasingly dependent in his daily routine

- to be cognitively moderately impaired

- to have a low risk of cardiovascular events

12. Would you deprescribe or decrease the dosage of one/several medication/s? (yes/no)

13. Which medication/s would you deprescribe or decrease?

- - Aspirin 100 mg once daily
  - Atorvastatin 40 mg once daily
  - Enalapril 10 mg once daily
  - Amlodipine 5 mg once daily
  - Paracetamol 1g three times a day
  - Tramadol 50 mg twice daily
  - Pantoprazole 20 mg once daily

14. Consider that Patient 2 now had a cardiovascular event in the past (e.g. myocardial infarction three years ago). Would you deprescribe or decrease the dosage of one/several medication/s? (yes/no)

15. Which medication/s would you deprescribe or decrease taking into account that Patient 2 had a cardiovascular event in the past (e.g. myocardial infarction three years ago)?

- - Aspirin 100 mg once daily
  - Atorvastatin 40 mg once daily
  - Enalapril 10 mg once daily
  - Amlodipine 5 mg twice daily
  - Paracetamol 1g three times a day
  - Tramadol 50 mg twice daily
  - Pantoprazole 20 mg once daily

***Case vignette 3***

In the following, there will be a case vignette.

After the case vignette, there will be a few questions asking you which medications you would deprescribe.

**Patient X**, 82 years of age:

**Social history**: retired carpenter*, lives together with his wife in a nursing home*

**General health**: *Patient X walks very little using a walker. Needs daily support for personal hygiene and getting dressed/undressed. Lack of spatial or temporal orientation. Unintended weight loss of 8kg in the past two months. MMSE 12/30.*

**Other diagnoses**: Chronic back pain, hypertension (last blood pressure measurements ranged from 130 to 140mmHG, systolic), non-smoker, no family history of cardiovascular events

**Laboratory values**: Dyslipidemia (LDL 3,8mmol/l), liver and kidney function are normal (taking into account the age of the patient), normal blood count

**Daily medication intake:**

Aspirin 100 mg once daily

Atorvastatin 40 mg once daily

Enalapril 10 mg once daily

Amlodipine 5 mg once daily

Paracetamol 1 g three times a day

Tramadol 50 mg twice daily

Pantoprazole 20mg once daily

**In this case vignette, you consider the patient:**

- to have strongly impaired physical functioning

- to be strongly dependent in his daily routine

- to be cognitively strongly impaired

- to have a low risk of cardiovascular events

16. Would you deprescribe or decrease the dosage of one/several medication/s? (yes/no)

17. Which medication/s would you deprescribe or decrease?

- - Aspirin 100 mg once daily
  - Atorvastatin 40 mg once daily
  - Enalapril 10 mg once daily
  - Amlodipine 5 mg twice daily
  - Paracetamol 1g three times a day
  - Tramadol 50 mg twice daily
  - Pantoprazole 20 mg once daily

18. Consider that this Patient had a cardiovascular event in the past (e.g. myocardial infarction three years ago). Would you deprescribe or decrease the dosage of one/several medication/s? (yes/no)

19. Which medication/s would you deprescribe or decrease taking into account that Patient X had a cardiovascular event in the past (e.g. myocardial infarction three years ago)?

- - Aspirin 100 mg once daily
  - Atorvastatin 40 mg once daily
  - Enalapril 10 mg once daily
  - Amlodipine 5 mg twice daily
  - Paracetamol 1g three times a day
  - Tramadol 50 mg twice daily
  - Pantoprazole 20 mg once daily

**C) Barriers and enablers to the willingness to deprescribe**

20. How important are the following ***patient characteristics*** for you when you deprescribe medications?

|  | Not important | Slightly important | Neutral | Important | Very important |
| --- | --- | --- | --- | --- | --- |
| Age |  |  |  |  |  |
| Life expectancy |  |  |  |  |  |
| Quality of life |  |  |  |  |  |
| Previous experiences with deprescribing |  |  |  |  |  |
| Expectations of the patient |  |  |  |  |  |
| Fear of potential negative health outcomes |  |  |  |  |  |
| Difficult communication |  |  |  |  |  |
| Expectation of relatives |  |  |  |  |  |

21. How important are the following criteria for you when you deprescribe medications?

|  | Not important | Slightly important | Neutral | Important | Very important |
| --- | --- | --- | --- | --- | --- |
| Existence of deprescribing guidelines |  |  |  |  |  |
| Existence of tools that facilitate deprescribing |  |  |  |  |  |
| Interprofessional communication (between GPs and other prescribing physicians) |  |  |  |  |  |
| Interprofessional collaboration (between GPs and other prescribing physicians) |  |  |  |  |  |
| Expenditure of time |  |  |  |  |  |
| Self-dispensation of medication in GP office |  |  |  |  |  |
| Benefit of a medication |  |  |  |  |  |
| Risk of a medication |  |  |  |  |  |

22. Are there any other factors that influence deprescribing from your point of view? (yes/no)

In your opinion, which other factors influence deprescribing?

**Remarks and comments**

23. Do you have any additional comments or remarks regarding deprescribing?

***Appendix 2.***

| **Table 1** Response rate by country. | | | | |
| --- | --- | --- | --- | --- |
| **Country** | **Survey language(s)** | **Number of GPs in sample** | **Number of replies receive** | **Response rate** |
| Slovenia | Slovenian | 352 | 38 | 10.80% |
| Latvia | Latvian | 1002 | 122 | 12.18% |
| Belgium | French, Dutch | 919 | 134 | 14.58% |
| Ireland | English | 113 | 20 | 17.70% |
| Luxembourg | French, Dutch, German | 100 | 21 | 21.00% |
| Netherlands | Dutch | 128 | 41 | 32.03% |
| Israel | English | 350 | 129 | 36.86% |
| Germany | German | 128 | 51 | 39.84% |
| Macedonia | Macedonian | 74 | 31 | 41.89% |
| Hungary | Hungarian | 587 | 248 | 42.25% |
| Austria | German | 80 | 36 | 45.00% |
| Estonia | Estonian | 52 | 24 | 46.15% |
| Sweden | Swedish | 113 | 53 | 46.90% |
| Spain | Spanish | 51 | 24 | 47.06% |
| France | French | 43 | 21 | 48.84% |
| Denmark | Danish | 58 | 29 | 50.00% |
| New Zealand | English | 78 | 42 | 53.85% |
| Switzerland | German, French | 288 | 157 | 54.51% |
| Czech Republic | Czech | 35 | 20 | 57.14% |
| Portugal | Portuguese | 72 | 43 | 59.72% |
| Romania | Romanian | 48 | 29 | 60.42% |
| Poland | Polish | 56 | 38 | 67.86% |
| Brazil | Portuguese | 87 | 62 | 71.26% |
| Greece | Greek | 70 | 50 | 71.43% |
| Italy | Italian | 43 | 31 | 72.09% |
| Finland | English | 31 | 23 | 74.19% |
| Ukraine | Ukrainian | 36 | 28 | 77.78% |
| United Kingdom | English | 30 | 25 | 83.33% |
| Bulgaria | Bulgarian | 41 | 36 | 87.80% |
| Bosnia and Herzegovina | Bosnian | 70 | 62 | 88.57% |
| Croatia | Croatian | 40 | 38 | 95.00% |
| **Average response rate** | **5175** | | **1706** | **52.52%** |

***Appendix 3. Results of sensitivity analyses***

| **Table 2** Analysis restricted to countries with a response rate >60% (number of countries in the analysis: 11): Percentage of general practitioners (GPs) deprescribing in case vignettes, sorted by GPs’ decisions to deprescribe at least one, two or three medications in the respective case vignette, patients’ level of dependency in activities of daily living, and patients’ history of cardiovascular disease (CVD) (n=361) | | | | | |
| --- | --- | --- | --- | --- | --- |
| Case vignette | Patients’ dependency level | Deprescribing decision | **Without** history of CVD (95% CI) | **With** history of CVD (95% CI) | Difference  (95% CI)^1^ |
| 1 | low  (living in own house, no help needed for activities of daily living) |  |  |  |  |
|  |  | min. 1 medication | 96.7 (94.8 – 98.6) | 84.4 (80.5 – 88.3) | 12.3 (8.0 – 16.7) |
|  |  | min. 2 medications | 91.1 (88.0 – 94.1) | 67.9 (62.8 – 73.0) | 23.0 (17.3 – 29.1) |
|  |  | min. 3 medications | 73.6 (68.9 – 78.3) | 33.0 (27.9 – 38.1) | 40.6 (33.6 – 47.5) |
| 2 | medium  (living in own house, some help needed for activities of daily living) |  |  |  |  |
|  |  | min. 1 medication | 94.5 (92.4 – 97.3) | 88.6 (85.0 – 92.1) | 6.3 (1.9 – 10.6) |
|  |  | min. 2 medications | 88.5 (84.9 – 66.2) | 71.2 (66.2 – 76.3) | 17.2 (11.0 – 23.4) |
|  |  | min. 3 medications | 67.6 (62.4 – 72.8) | 37.9 (32.5 – 43.4) | 29.7 (22.2 – 37.2) |
| 3 | high  (living in nursing home, help needed for nearly all activities of daily living) |  |  |  |  |
|  |  | min. 1 medication | 91.5 (88.3 – 94.7) | 88.6 (82.7 – 90.5) | 4.9 (0.0 - 9.9) |
|  |  | min. 2 medications | 86.8 (82.9 – 90.6) | 76.4 (71.5 – 81.2) | 10.4 (4.2 – 16.6) |
|  |  | min. 3 medications | 74.9 (70.0 – 79.9) | 51.0 (45.3 – 56.8) | 23.9 (16.3 – 31.5) |
| ^1^Two-sample test of proportions using variables | | | | | |
